# Floristic Composition and Diversity Along a Successional Gradient in Andean Montane Forests, Southwestern Colombia

**DOI:** 10.3390/plants15030389

**Published:** 2026-01-27

**Authors:** Víctor Alfonso Mondragón Valencia, Luis Gerardo Chilito, Carlos Edward Cabezas-Majín, Diego Jesús Macías Pinto

**Affiliations:** 1 Facultad de Ciencias Naturales Exactas y de la Educación, Doctorado en Ciencias Ambientales, Universidad del Cauca, Campus Tulcán, Popayán 190003, Colombia; 2 Grupo de Investigación en Ecología Tropical—UNIET, Programa de Ecología, Fundación Universitaria de Popayán, Popayán 190003, Colombia

**Keywords:** Andean montane forests, ecological succession, floristic composition, forest structure, natural regeneration, ecological restoration

## Abstract

Tropical Andean forests are biodiversity hotspots that have been transformed by anthropogenic activities, making ecosystem regeneration and restoration essential for their recovery. This study evaluated floristic composition, forest structure, and diversity in three land cover types within tropical Andean ecosystems: riparian forest (RF), natural regeneration (NR), and ecological restoration areas (RE). Vegetation was inventoried using standardized plots, recording species composition, diameter, and height. Basal area, size class distribution, and vertical structure were estimated. The Shannon Wiener and Simpson indices were evaluated. RF showed the highest structural complexity and basal area among the evaluated cover types, followed by ER, whereas NR showed the lowest values. NR showed the highest diversity values and a predominance of individuals in lower diameter and height classes, reflecting active recruitment and intermediate successional stages. Segment ER exhibited lower diversity and intermediate structural development, consistent with shorter recovery periods and limitations in restoration design. Overall, the integration of floristic, structural, and diversity attributes indicates distinct successional trajectories, conditioned by land-use history, disturbance intensity, and environmental heterogeneity. These findings highlight the great potential for natural regeneration under reduced anthropogenic pressure and emphasize the need to integrate passive and active restoration strategies to enhance biodiversity and resilience in Andean tropical forests.

## 1. Introduction

Biological diversity is fundamental to the stability, functioning, and long-term persistence of terrestrial ecosystems. It underpins key ecological processes and provides ecosystem services essential for human well-being [1]. In this sense, understanding the structure and composition of biological communities is crucial for designing effective conservation strategies and guiding ecosystem management, especially in regions where biodiversity loss is accelerating as a result of anthropogenic pressures [2]. Floristic inventories and vegetation studies play a central role, as they provide baseline information on species composition, diversity patterns, and structural attributes of ecosystems, contributing to a better understanding of ecological processes and the definition of conservation priorities [3].

The tropical Andean ecosystems of Colombia are recognized as one of the most important centers of biodiversity on the planet, characterized by high levels of species richness and endemism [4,5]. This extraordinary diversity is the result of a complex geological and evolutionary history, coupled with marked environmental heterogeneity generated by topography, climatic variability, and biogeographic connectivity [6]. However, these ecosystems have also been intensely transformed by human activities, as they form the basis of Colombia’s agricultural, urban, and cultural development. Consequently, a significant proportion of Andean forests has been fragmented or replaced, leading to an accelerated reduction in forest cover and biodiversity, primarily due to deforestation, land-use change, and the expansion of the agricultural frontier. Landscape fragmentation is currently considered one of the main drivers of biodiversity loss in the Andes [7].

Given this scenario of high disturbance, the study of ecological succession becomes especially relevant for understanding ecosystem resilience, recovery trajectories, and long-term dynamics. Secondary succession involves progressive changes in species composition, structural complexity, and functional processes, which can eventually lead to conditions similar to those before the disturbance or to the formation of distinct ecological states [8]. Analyzing these successional trajectories is fundamental to assessing the potential of natural regeneration as a passive restoration mechanism, as well as the effectiveness of active ecological restoration strategies, particularly in tropical montane forests, where recovery processes can be slow and highly dependent on the environmental context and land use history [9].

Despite the ecological importance of Andean montane forests, comparative studies that comprehensively assess floristic composition, diversity, and vegetation structure along successional gradients are still limited [10,11]. While riparian vegetation has been widely recognized as a key component influencing ecosystem integrity and hydrological functioning in tropical landscapes, it has often been addressed from functional or integrative assessment frameworks rather than through detailed analyses of plant community composition and structure [12]. Consequently, riparian forests are still underrepresented in comparative studies that include conserved reference ecosystems, areas undergoing natural regeneration, and sites subject to active ecological restoration, despite their role as biodiversity reservoirs, ecological corridors, and valuable reference systems for restoration planning.

In this context, the present study contributes to the knowledge of floristic composition, diversity, and structural attributes of different vegetation cover types along a successional gradient in the Las Piedras River basin, located in Popayán, Cauca, in southwestern Colombia. This landscape is a heterogeneous mosaic, shaped by historical land use and current management practices, and includes preserved riparian forests (RF), areas of natural regeneration (NR), and sites undergoing active ecological restoration (ER) [13]. The objective of this study was to compare floristic diversity and vegetation structure among these cover types, as well as to evaluate their successional potential and ecological functionality. The results aim to contribute to the understanding of forest recovery processes in Andean montane ecosystems and provide input for the design of conservation and restoration strategies in fragmented tropical landscapes.

## 2. Materials and Methods

### 2.1. Study Area

**Figure 1:**
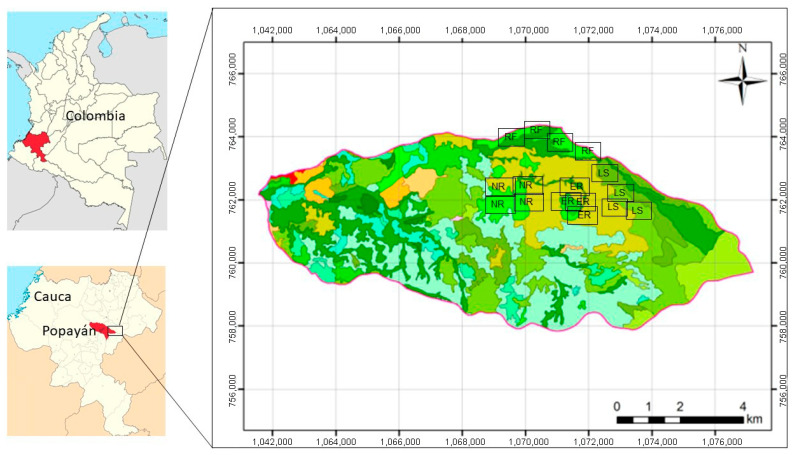
Location of the study area: Coordinates are expressed in UTM (meters), Datum WGS84, Zone 18 N.

The study was conducted in the Las Piedras River basin, located in the intertropical equatorial Andes in southwestern Colombia, within the municipality of Popayan, Cauca (2°21′35″ N, 76°33′10″ W). The basin covers approximately 58 km^2^ and spans an altitudinal range from 1980 to 3820 m a.s.l., encompassing sub-Andean and Andean forest formations (Figure 1).

The landscape is predominantly mountainous, with steep slopes in the upper basin (35–98%), moderate slopes in the middle zone (16–35%), and gentle slopes in the lower zone (3–15%). The climate corresponds to equatorial montane conditions, with mean annual temperatures ranging from 10.4 to 18.4 °C and a pronounced seasonality in precipitation, characterized by a wet period from October to May and a relatively dry season from June to September.

Soils are mainly Andisols derived from volcanic ash, with loamy to clay-loam textures, good drainage, weak structure, and strongly acidic conditions. These environmental characteristics, combined with pronounced topographic heterogeneity, promote high plant diversity and the development of a mosaic of forest covers along the successional gradient [14].

### 2.2. Vegetation Sampling

Three vegetation cover types representative of a successional gradient within the watershed were evaluated: riparian forest (RF), natural regeneration (NR), and ecological restoration (ER). The RF corresponds to a well-preserved primary forest that has been protected for several generations and has not been subjected to logging or major anthropogenic disturbances, thus serving as a reference ecosystem. ER areas have been designated for restoration purposes since approximately 1991 and comprise about 45 ha. These areas have been managed under long-term community-based restoration efforts, with no intensive silvicultural treatments applied. A portion of the ER (approximately 15 ha) was affected by a fire event and subsequently underwent natural regeneration, allowing spontaneous recovery processes to dominate. In each vegetation cover, six replicate linear transects measuring 50 × 4 m (200 m^2^) were established, resulting in a total sampled area of 0.12 ha per cover type. The study followed a comparative observational design across spatially separated cover types within the same landscape unit. Vegetation cover types were distributed within the same watershed and along comparable altitudinal ranges and environmental conditions. To control background heterogeneity, transects were established using consistent sampling criteria within each cover type, avoiding spatial overlap and maintaining a minimum distance of 30 m between transects. This approach allowed for robust comparisons among vegetation covers while minimizing the influence of large-scale environmental variability.

In each transect, all woody individuals with a diameter at breast height (DBH) ≥ 5 cm were recorded. For each individual, the species, circumference at breast height (CBH), stem height, and total height (m) were recorded, following standard methodologies for forest structure studies [15]. In areas dominated by grasslands, the proportion of individuals per unit area was estimated. Botanical specimens were collected, pressed, and processed using standard herbarium techniques. Voucher specimens were deposited in the Álvaro Fernández Pérez Herbarium (AFP) of the Fundación Universitaria de Popayán.

### 2.3. Taxonomic Identification

The taxonomic identification of the species was carried out using specialized keys, regional botanical literature, and digital resources, including keys for Rubiaceae and Melastomataceae of Colombia [16,17], as well as online databases such as World Flora Online (WFO), Useful Plants of Colombia (ColPlantA), Tropicos, Plants of the World Online (POWO), additionally, direct comparison was made with specimens deposited in the herbaria AFP (Fundación Universitaria de Popayán) and CAUP (Universidad del Cauca).

### 2.4. Structural and Diversity Analyses

Vegetation structural attributes were quantified based on the field data. Tree density was calculated as the number of individuals per hectare (individuals ha^−1^), extrapolated from the sampled area. Basal area (m^2^ ha^−1^) was estimated as the sum of the cross-sectional area of all recorded stems at DBH [18].

Horizontal structure was analyzed by grouping individuals into diameter classes, while vertical structure was assessed using height classes. Class intervals for both diameter and height were defined using Sturges’ rule to facilitate comparison among vegetation covers. Distributions were expressed as absolute frequencies and as proportions (%) of the total number of individuals per cover type [19].

Alpha diversity was evaluated using the Shannon–Wiener diversity index (H′), which accounts for species richness and evenness, and the Simpson index (λ), which reflects dominance patterns. Lower Simpson values were interpreted as higher diversity [20].

To account for the sensitivity of diversity indices to sampling effort and stem density, species accumulation curves and individual-based rarefaction analyses were performed for each vegetation cover. Rarefaction curves were constructed using the cumulative number of individuals to allow standardized comparisons of species richness among cover types. Observed and rarefied species richness were used to complement Shannon and Simpson diversity indices [21].

Floristic beta-diversity among vegetation covers was quantified using pairwise comparisons based on species presence–absence data. Overall dissimilarity was calculated using the Sørensen index and subsequently partitioned into turnover and nestedness components following the framework proposed by [22]. Species turnover represents compositional differences driven by species replacement between covers, whereas nestedness reflects differences associated with species loss or gain. The relative contribution of each component was expressed as a percentage of total beta-diversity for each pairwise comparison (ER–NR, RF–ER, and RF–NR).

### 2.5. Data Processing, Software, and Visualization

The analyses focused on the descriptive and comparative evaluation of structural and diversity attributes among vegetation cover types. Given the observational nature of the study and the spatial separation of the cover types, results were interpreted based on observed patterns rather than formal hypothesis testing. No inferential statistical tests were applied, and variability among transects is reported descriptively to characterize within-cover structural consistency.

All statistical analyses and graphical representations were performed using R software version 4.5.1. Data manipulation and calculation of structural and diversity metrics were conducted using the packages dplyr and vegan [23], while figures were produced using ggplot2 [24].

Vegetation profiles illustrating vertical stratification patterns were generated using Python code in Blender version 5.0 [25], based on field-measured height and DBH data. These profiles represent schematic and idealized depictions of the dominant structural characteristics of each vegetation cover, rather than exact spatial reconstructions of individual transects.

Exploratory analyses and cross-validation of diversity indices were additionally supported using PAST software version 2.17 [26].

Floristic beta-diversity analyses were conducted in R version 4.5.1 using the package betapart [27].

## 3. Results

### 3.1. Species Composition

The floristic composition recorded across the studied vegetation covers revealed a clear dominance of a limited number of plant families in the study area: Melastomataceae was the most representative family, with six genera and nine species, followed by Asteraceae (six genera and seven species) and Lauraceae (five genera and six species). Other families with a notable contribution included Clusiaceae (three genera and five species), Escalloniaceae (two genera and three species), as well as Phyllanthaceae and Solanaceae, each represented by two genera and two species.

**Figure 2:**
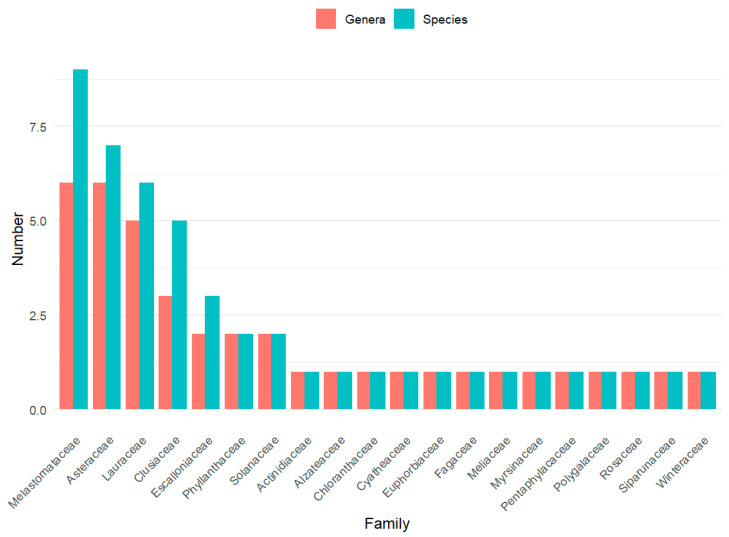
Representative families by genera and species.

Most of the remaining families showed low representation, typically with one or two genera and a single species per family, reflecting a common pattern in Andean montane plant communities, where floristic diversity is unevenly distributed among taxonomic groups (Figure 2).

The floristic composition varied markedly among the evaluated vegetation covers (Table 1), reflecting differences in successional stage and land-use history. RF were characterized by the presence of late-successional and forest-associated species such as *Aniba perutilis*, *Cedrela odorata*, *Juglans neotropica*, *Quercus humboldtii*, and *Weinmannia tomentosa*, indicating more structurally developed conditions. ER showed a mixed species composition, including taxa shared with riparian forests (e.g., *Meriania speciosa*, *Myrcia popayanensis*, *Oreopanax incisus*) and species typical of secondary or disturbed environments, suggesting intermediate successional stages. In contrast, NR were dominated by pioneer and early-successional species, particularly from Asteraceae and Poaceae, such as *Ageratina theifolia*, *Baccharis* sp., *Chromolaena ivifolia*, and *Cenchrus purpureus*. Several species occurred in more than one vegetation cover, indicating floristic overlap along the successional gradient.

**Figure 3:**
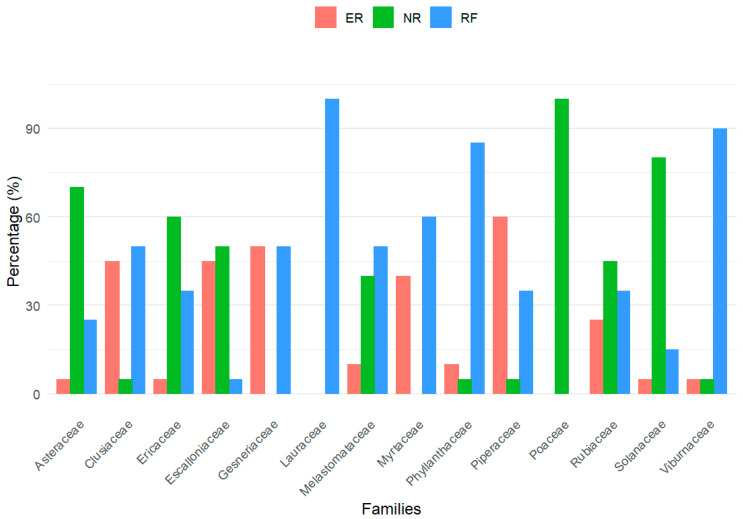
Representative families by coverage (%).

The relative contribution of plant families showed contrasting patterns among vegetation covers (Figure 3). RF was characterized by families typical of mature Andean forests, particularly Lauraceae, Viburnaceae, Phyllanthaceae, and Myrtaceae, whereas ecological restoration sites (ER) exhibited an intermediate pattern, with higher representation of families such as Gesneriaceae and Piperaceae. In contrast, natural regeneration areas (NR) were characterized by a greater representation of early-successional and herbaceous families, including Poaceae, Solanaceae, Asteraceae, and Ericaceae, reflecting floristic differences along the successional gradient.

### 3.2. Forest Structure of Land Uses

Forest structural attributes differed among the evaluated plant covers (Table 2). RF and ER showed comparable basal area values, with means of 15.75 ± 1.03 m^2^ ha^−1^ and 16.33 ± 1.29 m^2^ ha^−1^, respectively, whereas NR exhibited substantially lower basal area (6.08 ± 1.45 m^2^ ha^−1^). A similar pattern was observed for tree density, with higher values recorded in RF (1138 ± 6.49 trees ha^−1^) and ER (1085 ± 5.57 trees ha^−1^) compared to NR (873 ± 3.87 trees ha^−1^). These results indicate clear structural differences among vegetation covers, consistent with their contrasting successional conditions.

**Figure 4:**
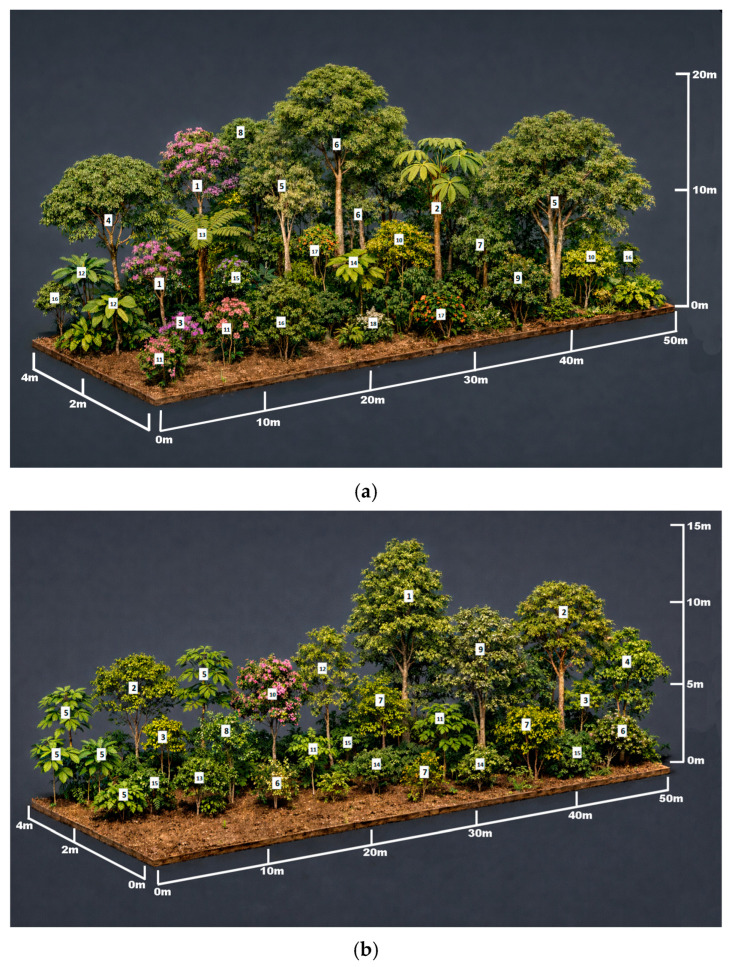

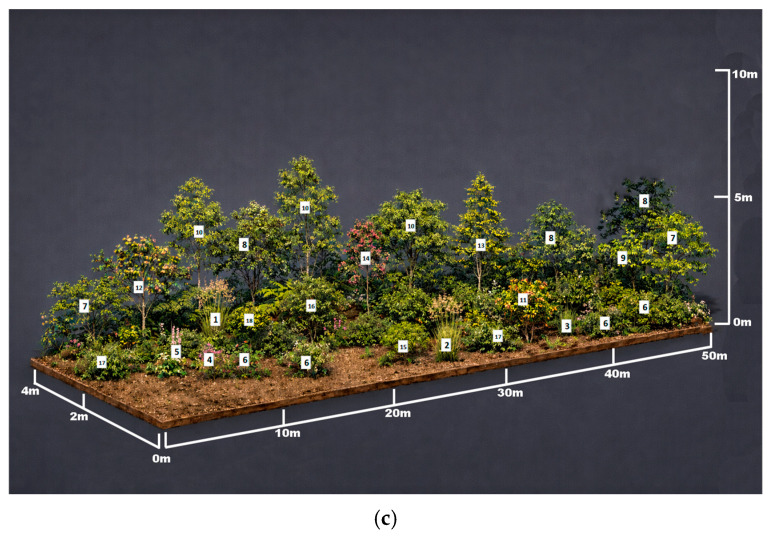
Idealized profiles of the studied vegetation covers: (**a**) RF: 1. *Axinaea macrophylla*; 2. *Cecropia peltata*; 3. *Andesanthus lepidotus*; 4. *Quercus humboldtii*; 5. *Aniba perutilis*; 6. *Cedrela odorata*; 7. *Hieronyma macrocarpa*; 8. *Ocotea oblonga*; 9. *Miconia notabilis*; 10. *Palicourea angustifolia*; 11. *Chaetogastra mollis*; 12. *Piper crassinervium*; 13. *Cyathea* sp.; 14. *Siparuna echinata*; 15. *Myrcia popayanensis*; 16*. Hedyosmum cumbalense*; 17. *Cinchona pubescens*; 18. *Monnina salicifolia*, (**b**) ER: 1. *Alzatea verticillata*; 2*. Clusia multiflora*; 3. *Columnea* cf. *Sanguinea*; 4. *Hedyosmum cumbalense*; 5. *Oreopanax incisus*; 6. *Aparisthmium cordatum*; 7. *Palicourea thyrsifolia*; 8. *Palicourea angustifolia*; 9. *Escallonia paniculata*; 10. *Meriania speciosa*; 11. *Morella pubescens*; 12. *Myrcia popayanensis*; 13. *Myrcianthes rhopaloides*; 14*. Myrsine coriacea;* 15*. Piper aduncum*, (**c**) NR: 1. *Chusquea* sp.; 2. *Cortaderia nitida*; 3. *Holcus lanatus*; 4. *Lolium multiflorum*; 5. *Monochaetum* sp.; 6. *Rhynchospora nervosa*; 7. *Vaccinium meridionale Sw.*; 8*. Ageratina theifolia*; 9*. Baccharis* sp.; 10. *Bejaria resinosa*; 11. *Miconia notabilis*; 12. *Cinchona pubescens*; 13*. Chaetogastra grossa*; 14. *Chaetogastra mollis*; 15. *Chromolaena ivifolia*; 16. *Fuchsia caucana*; 17. *Galium hypocarpium*; 18. *Gynoxys* sp.

The vegetation in RF shows a well-defined vertical stratification with a closed canopy reaching approximately 20 m, a developed subcanopy, and a dense understory, indicating a structurally mature forest (Figure 4a). ER exhibits intermediate structural complexity, with a heterogeneous canopy (up to ~15 m), mixed planted and naturally regenerated individuals, and an incipient but continuous understory (Figure 4b). In contrast, NR is characterized by a simpler structure, dominated by low- to medium-height vegetation (≤10 m), a discontinuous canopy, and greater light penetration, typical of early successional stages (Figure 4c). Taken together, these vegetation profiles reflect a clear gradient of increasing structural complexity from NR to ER and RF (Figure 4).

### 3.3. Diameter Distribution

The diameter class distribution highlights clear differences in the structural development of the studied vegetation covers. In all cases, individuals are predominantly concentrated in the lower diameter classes (DBH < 0.30 m), indicating active regeneration processes and a prevalence of young trees. However, the relative contribution of intermediate and larger diameter classes varies among plant covers, suggesting contrasting successional stages and structural complexity in RF, ER and NR (Table 3).

### 3.4. Altimetric Distribution

The height class distribution further illustrates differences in vertical structure among vegetation covers. In all covers, individuals were predominantly concentrated in the lower height classes (≤10 m), indicating the prevalence of juvenile and subcanopy strata. RF exhibited a higher proportion of individuals in intermediate and upper height classes, reflecting greater vertical stratification and structural development. ER showed an intermediate pattern, whereas NR were strongly dominated by low-stature individuals, consistent with early successional stages and simpler vertical structure (Table 4).

### 3.5. Ecological Indices

Alpha diversity differed among vegetation covers as reflected by the Shannon and Simpson indices (Table 5). NR exhibited the highest diversity values (Shannon = 2.89; Simpson = 0.79), followed by RF (Shannon = 2.45; Simpson = 0.86), whereas ER showed comparatively lower diversity (Shannon = 2.03; Simpson = 0.79). These patterns indicate differences in species richness and evenness among covers, providing a quantitative basis for comparing floristic diversity along the successional gradient.

### 3.6. Species Richness, Rarefaction, and Sampling Completeness

**Figure 5:**
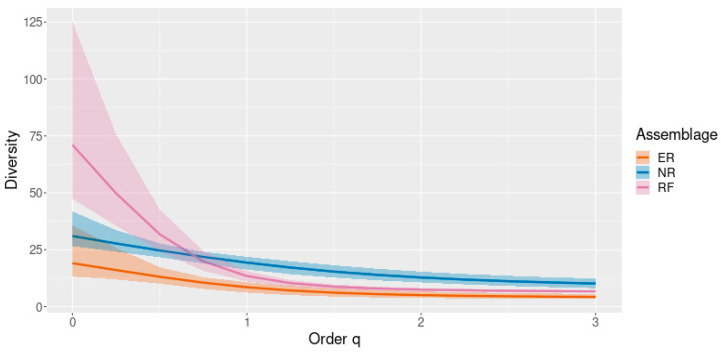
Diversity profiles based on Hill numbers (q = 0–3) for the different vegetation cover types. RF: riparian forest; ER: ecological restoration; NR: natural regeneration.

Diversity was also assessed using Hill numbers (orders q = 0–3) to evaluate richness and abundance-weighted diversity across vegetation covers (Figure 5). This approach allows comparisons that are less sensitive to differences in sample size and stem density.

**Figure 6:**
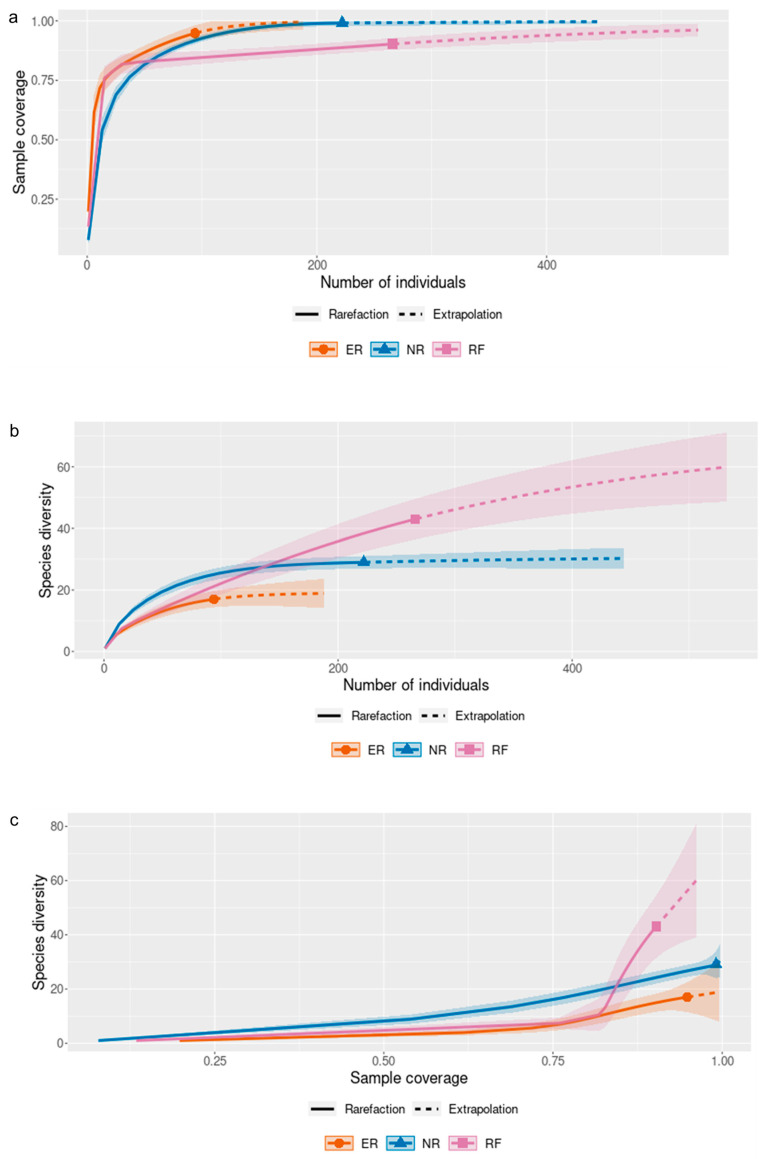
Individual-based rarefaction and extrapolation analyses of species richness across vegetation cover types: (**a**) sample coverage as a function of the number of individuals; (**b**) species richness as a function of the number of individuals; (**c**) species richness as a function of sample coverage. Solid lines represent rarefaction curves and dashed lines represent extrapolation curves. Shaded areas indicate 95% confidence intervals. RF: riparian forest; ER: ecological restoration; NR: natural regeneration.

Individual-based rarefaction and extrapolation curves revealed clear differences in species richness patterns among vegetation cover types when standardized by sampling effort (Figure 6). Sample coverage increased rapidly with the number of individuals in all covers, reaching values close to 1.0, indicating high sampling completeness, particularly in ER and NR (Figure 6a). Rarefaction curves based on the number of individuals showed that RF achieved the highest observed and extrapolated species richness, followed by NR, whereas ER consistently exhibited lower richness across comparable sampling effort (Figure 6b). Extrapolation suggests that additional sampling would likely yield a greater increase in species richness in RF than in the other covers. When species richness was evaluated as a function of sample coverage, RF maintained higher richness values across the full range of coverage, while NR displayed intermediate richness and ER the lowest values (Figure 6c). These patterns indicate that differences in species richness among vegetation covers are not solely driven by sampling effort or stem density, but reflect underlying differences in community composition along the successional gradient.

**Figure 7:**
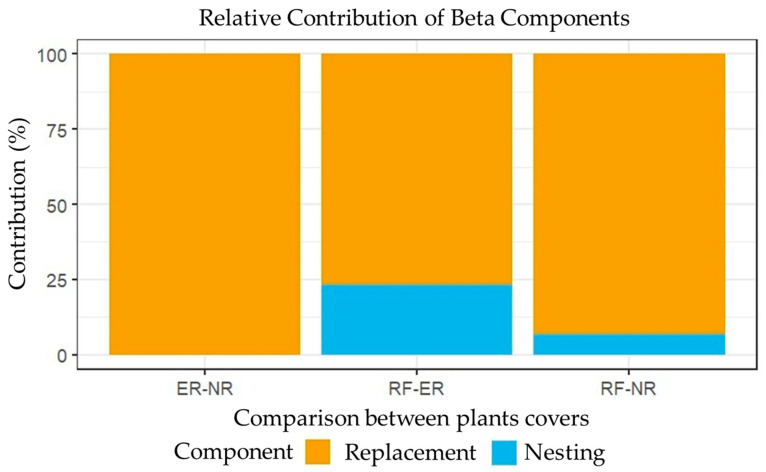
Relative contribution of turnover and nestedness components to beta-diversity among vegetation covers along the successional gradient.

Beta-diversity partitioning revealed that species turnover was the dominant component differentiating vegetation covers along the successional gradient (Figure 7). Pairwise comparisons showed that the ER–NR and RF–NR contrasts were largely driven by species replacement, indicating distinct floristic assemblages among these covers. In contrast, the RF–ER comparison exhibited a higher relative contribution of the nestedness component, suggesting partial compositional overlap between restored areas and riparian forests.

## 4. Discussion

### 4.1. Floristic Composition and Forest Structure

The floristic composition of the different vegetation cover types (Table 1; Figure 3) reveals contrasting patterns of species composition and dominance that are consistent with differences in disturbance history and degrees of structural development. RF showed a greater representation of woody species and families typical of mature Andean montane forests, generally associated with shade-tolerant, slow-growing, and longer-lived species, indicating floristically more consolidated communities in structural and compositional terms. These characteristics are consistent with relatively stable local environmental conditions and well-developed forest structure, although direct measurements of ecosystem functioning were beyond the scope of this study [28,29]. In contrast, the NR areas showed high species richness dominated by pioneer and early successional species, as well as by families with ecological strategies geared toward rapid establishment and efficient dispersal, reflecting active recruitment and turnover typical of structurally young secondary forests [30]. Meanwhile, the ER areas exhibited lower species richness and a more homogeneous composition, primarily influenced by the time elapsed since the intervention and subsequent natural regeneration processes, resulting in an intermediate floristic and family composition, with different states of vegetation cover development [31]. Taken together, these patterns suggest that the observed floristic, structural, and functional differences are not random but rather reflect contrasting structural conditions and management histories, rather than fully resolved successional stages or functional trajectories.

The structural comparison between the land cover types (Table 2) showed that the RF exhibited the greatest structural complexity, reflected in high basal area values, high density, and a more balanced distribution of individuals across diameter and height classes, indicating greater structural development. These attributes are commonly used as proxies of forest structural complexity but should not be interpreted as direct evidence of ecosystem stability or functioning. In contrast, the ER showed an intermediate structural condition, dominated by young, growing individuals, characteristic of the initial stages of active restoration, while the natural regeneration (NR) presented a smaller basal area and a high concentration of individuals in lower classes, indicating an early phase of structural development. This pattern aligns with studies demonstrating that, during tropical secondary succession, basal area progressively increases as a result of diameter growth and biomass accumulation, gradually approaching the values observed in mature forests, in parallel with an increase in the structural and functional complexity of the ecosystem [32]. However, basal area recovery does not occur uniformly, as land-use history can modify the rate and trajectory of structural development, leading to contrasting structural patterns even in forests of similar age. In this sense, the intensity and duration of previous land uses influence the convergence toward structures characteristic of mature forests and have direct implications for planning restoration strategies [33].

When compared with other studies conducted in Andean montane forests of Colombia and the Tropical Andes, the floristic and structural attributes observed in this study fall within the ranges commonly reported for similar ecosystems. The dominance of families such as Melastomataceae, Lauraceae, Myrtaceae, Asteraceae, and Rubiaceae has been widely reported in sub-Andean and Andean forests across the Tropical Andes, including Colombia, Ecuador, Peru, and Bolivia, reflecting shared biogeographic histories and strong environmental filtering associated with elevation, climate, and soil conditions [10,34]. Likewise, basal area values recorded in riparian forests and ecological restoration sites (approximately 15–16 m^2^ ha^−1^) are comparable to those reported for secondary and mid-successional Andean forests, where basal area commonly falls within a broad range (approximately 12–30 m^2^ ha^−1^), depending on forest age, land-use history, and disturbance intensity [35,36]. Furthermore, the predominance of individuals in smaller diameter and height classes, together with Shannon diversity values between 2.0 and 3.0, is characteristic of structurally developing tropical montane forests, particularly during intermediate stages where pioneer and shade-tolerant species coexist. This coexistence promotes high structural heterogeneity and diversity, reflecting active recruitment and coexistence of contrasting life-history strategies, rather than advanced successional or functionally stable states in tropical secondary forests [37,38,39]. These comparisons indicate that the studied stands are consistent with broader structural and compositional patterns reported for Andean forests in Colombia and across South America, supporting their relevance for understanding regional successional dynamics and informing restoration strategies in tropical mountain landscapes.

Taken together, the integration of floristic composition patterns (Table 1; Figure 3) and forest structure (Table 2) confirms that the evaluated land covers represent distinct structural and compositional conditions shaped by land-use history and environmental heterogeneity, where environmental heterogeneity, land-use history, and disturbance dynamics decisively condition the configuration of plant communities. The greater floristic and structural complexity observed in the RF is consistent with forests exhibiting higher vertical and horizontal complexity. In contrast, the NR and ER exhibit compositional and structural arrangements characteristic of early and intermediate successional stages of structural development. These differences reflect the close interdependence among structure and composition in the tropical Andes, where long-term successional processes define contrasting trajectories of forest development and determine ecosystem complexity, stability, and resilience at the landscape scale [33,40].

### 4.2. Diversity, Ecological Succession and Implications for Restoration

The calculated ecological indices reveal marked contrasts in diversity and dominance patterns among the evaluated land cover types (Table 4). The NR (Normal Forest) showed the highest diversity values according to the Shannon (H′) and Simpson (λ) indices, indicating a highly heterogeneous community with low dominance, a pattern commonly observed in structurally developing secondary forests, where the coexistence of pioneer, early secondary, and shade-tolerant species increases the structural and functional complexity of the system [41,42]. Although the RF (Fairy Forest) recorded the greatest overall floristic richness (Table 1), its diversity values were intermediate, reflecting a greater dominance of species typical of advanced successional stages, associated with canopy closure and an intensification of competition for resources, without implying a reduction in ecosystem stability [43]. In contrast, the ER areas showed the lowest values of diversity and evenness, consistent with their shorter recovery time and the influence of the restoration design on the initial floristic composition; in these systems, the increase in diversity occurs progressively and depends strongly on landscape connectivity, the availability of propagules and the action of dispersers, factors that condition the convergence towards more diverse and functionally complex communities, factors that condition compositional outcomes but were not directly quantified in this study [44]. Together, these patterns indicate that diversity and dominance dynamics are tightly linked to successional stage and management history, but in tropical Andean forests, they are further shaped by strong environmental heterogeneity associated with steep elevational gradients, complex topography, and pronounced microclimatic variation, which promote divergent ecological trajectories across the landscape [34].

From an ecological restoration perspective in tropical Andean forests, the integration of the results for floristic composition (Table 1), forest structure (Table 2), diversity (Table 4), and vegetation profiles (Figure 4) indicates that each land cover represents a distinct structural outcome, strongly influenced by land-use history, environmental heterogeneity, and the disturbance dynamics characteristic of these mountain ecosystems. RF can be considered a structural and compositional reference, playing a key role in microclimatic regulation, biodiversity conservation, and maintaining ecological processes at the landscape scale. NR highlights the high potential of passive restoration in the tropical Andes when anthropogenic pressures are reduced, favoring spontaneous processes of recruitment, diversification, and functional recovery. For their part, ER areas show that human intervention can accelerate the initial recovery of forests in degraded Andean contexts, provided it is based on adaptive management approaches that integrate species diversity, landscape connectivity, and ecological facilitation processes. Taken together, these results reinforce the need to combine passive and active strategies to restore biodiversity, ecosystem services, and the resilience of tropical mountain forests, priority ecosystems for conservation in the Andean region [45]. Furthermore, evidence from Andean forests indicates that natural regeneration after severe disturbances plays a key role in the progressive recovery of soil ecosystem services at different time scales, while traditional ecological knowledge can enhance these processes by guiding restoration practices more closely aligned with local dynamics, especially in Andean cloud forest ecosystems [46,47].

Overall, the diversity patterns revealed by the Hill numbers and rarefaction analyses support the interpretation of successional differentiation among vegetation communities (Figure 5 and Figure 6). The higher effective diversity observed in riparian forests reflects more consolidated communities with greater evenness and functional balance (Figure 5), a pattern well captured by diversity profiles based on Hill numbers, which integrate species richness and dominance into a unified framework [48]. In contrast, natural regeneration sites exhibit high species turnover but increased dominance by a reduced set of species, a characteristic feature of early to intermediate successional stages [49]. The lower diversity values recorded in ecological restoration areas suggest that, despite active intervention, these systems may require longer recovery periods or more heterogeneous planting designs to approach the diversity structure of more advanced forest conditions, a pattern widely reported for restored tropical forests and further supported by individual-based rarefaction analyses that control for sampling effort and completeness [50].

Complementarily, the partitioning of beta-diversity provided additional insight into the mechanisms underlying compositional differences among vegetation covers along the successional gradient (Figure 7). Species turnover was the dominant component in most pairwise comparisons, particularly between ER–NR and RF–NR, indicating that successional differentiation is primarily driven by species replacement rather than simple species loss or gain, a pattern commonly associated with strong ecological gradients and contrasting community assembly processes [51]. In contrast, the higher relative contribution of nestedness observed in the RF–ER comparison suggests partial compositional overlap, consistent with restoration trajectories in which early restored communities retain a subset of species characteristic of more mature forest conditions. This pattern aligns with conceptual frameworks that integrate community succession and assembly paradigms in ecological restoration, where restored ecosystems follow multiple, non-linear trajectories rather than converging rapidly toward a single reference state [52]. Together, these patterns highlight that both species replacement and nestedness contribute to shaping floristic composition in tropical Andean forests, depending on successional context and restoration strategy.

Taken together, the integration of multiple ecological dimensions in this study demonstrates that, in tropical Andean forests, diversity patterns, forest structure, and ecosystem functioning are determined by closely coupled successional processes operating at multiple spatial and temporal scales. The contrasts observed between land cover types reflect not only differences in disturbance history and management strategies but also the inherent environmental heterogeneity of Andean mountain landscapes, highlighting the need for local context-specific restoration approaches that align with the unique ecological dynamics of these ecosystems.

## 5. Conclusions

The results of this study indicate that floristic composition, diversity, and forest structure in tropical Andean forests respond to land-use history, disturbance intensity, and the environmental heterogeneity characteristic of mountainous landscapes. The differences observed between RF, NR, and ER reflect contrasting structural and compositional conditions that are expressed both in species dominance and in the structural and functional complexity of plant communities.

RF is consolidated as a reference ecosystem due to its greater structural and functional maturity, while NR demonstrates the high potential of passive processes for the recovery of diversity and ecosystem functioning when anthropogenic pressures are reduced. ER, for its part, shows that active interventions can accelerate the initial stages of recovery, although its convergence toward more complex states depends on the implementation of adaptive management approaches that integrate species diversity, landscape connectivity, and local ecological processes.

Overall, this study highlights that the effective restoration of tropical Andean forests requires differentiated and contextual strategies, based on an understanding of successional pathways and environmental heterogeneity. The combination of passive and active restoration emerges as a key strategy for strengthening biodiversity, ecosystem services, and the resilience of these mountain ecosystems, which are considered a priority for conservation in the face of global change.

## Figures and Tables

**Table 1:** General list of recorded species and their relationship with the studied land cover.

Family	Species	RF	ER	NR
Actinidiaceae	*Saurauia scabra* (Kunth) D.Dietr.	X		
Alstroemeriaceae	*Bomarea patinii* Baker	X		X
Alzateaceae	*Alzatea verticillata* Ruiz & Pav		X	
Araliaceae	*Oreopanax incisus* (Willd. ex Schult.) Decne. & Planch.	X	X	
Asteraceae	*Ageratina theifolia* (Benth.) R.M.King & H.Rob.			X
	*Baccharis* sp.			X
	*Cirsium vulgare* (Savi) Ten	X		
	*Gynoxys* sp.			X
	*Smallanthus pyramidalis* (Triana) H.Rob	X		X
	*Chromolaena ivifolia* (L.) R.King & H.Rob.			X
Chloranthaceae	*Hedyosmum cumbalense* H.Karst.	X	X	
Clusiaceae	*Clusia alata* Planch. & Triana	X		
	*Clusia multiflora* Kunth		X	
Cunoniaceae	*Weinmannia tomentosa* L.f.	X		
Cyatheaceae	*Cyathea* sp.	X		
Cyperaceae	*Rhynchospora nervosa* (Vahl) Boeckeler			X
Ericaceae	*Bejaria resinosa* Mutis ex L.fil.			X
	*Vaccinium meridionale* Sw.	X		X
Escalloniaceae	*Escallonia myrtilloides* L.fil.			X
	*Escallonia paniculata* (Ruiz & Pav.) Roem. & Schult.		X	
Euphorbiaceae	*Aparisthmium cordatum* (A.Juss.) Baill		X	
Fabaceae	*Inga densiflora* Benth.	X		
Fagaceae	*Quercus humboldtii* Bonpl.	X		
Gesneriaceae	*Columnea* cf. *sanguinea* (Pers.) Hanst.		X	
	*Kohleria warszewiczii* (Regel) Hanst.	X		
Juglandaceae	*Juglans neotropica* Diels	X		
Lauraceae	*Aniba perutilis* Hemsl.	X		
	*Nectandra mollis* (Kunth) Nees	X		
	*Ocotea oblonga* (Meisn.) Mez	X		
Melastomataceae	*Andesanthus lepidotus* (Humb. & Bonpl.) P.J.F.Guim. & Michelang.	X		
	*Axinaea macrophylla* Triana	X		
	*Chaeotogastra grossa* (L.fil.) P.J.F.Guim. & Michelang.	X		X
	*Chaetogastra mollis* (Bonpl.) DC.	X		X
	*Meriania nobilis* Triana	X		
	*Meriania speciosa* (Bonpl.) Naudin		X	
	*Miconia notabilis* Triana	X		X
	*Miconia theaezans* (Bonpl.) Cogn.	X		X
	*Monochaetum* sp.			X
Meliaceae	*Cedrela odorata* L.f.	X		
Myricaceae	*Morella pubescens* (Humb. & Bonpl. ex Willd.) Wilbur		X	
Myrsinaceae	*Myrsine coriacea* (Sw.) R.Br. ex Roem. & Schult.	X	X	
Myrtaceae	*Myrcia popayanensis* Hieron	X	X	
	*Myrcianthes fragans* (Sw.) McVaugh	X		
	*Myrcianthes rhopaloides* (Kunth) Mc Vaugh	X	X	
Onagraceae	*Fuchsia caucana* P.E.Berry			X
Pentaphylacaceae	*Freziera reticulata* Bonpl.	X		
Phyllanthaceae	*Hieronyma macrocarpa* Müll. Arg.	X		
	*Phyllanthus salviifolius* Kunth	X		
Pinaceae	*Pinus radiata* D.Don			X
Piperaceae	*Piper aduncum* L.		X	
	*Piper crassinervium* Kunth.	X	X	
Poaceae	*Cenchrus purpureus* (Schumach.) Morrone			X
	*Chusquea* sp.			X
	*Cortaderia nitida* (Kunth) Pilg.			X
	*Holcus lanatus* L.			X
	*Lolium multiflorum* Lam.			X
Polygalaceae	*Monnina salicifolia* Ruiz & Pav.	X		
Primulaceae	*Stylogyne* sp.		X	
Rosaceae	*Rubus bogotensis* Kunth			X
Rubiaceae	*Cinchona pubescens* Vahl	X		X
	*Coccocypselum lanceolatum* (Ruiz & Pav.) Pers.			X
	*Elaeagia pastoensis* L.E. Mora			X
	*Galium hypocarpium* (L.) Endl. ex Griseb.			X
	*Ladenbergia oblongifolia* (Humb. ex Mutis) L.Andersson	X		
	*Palicourea thyrsifolia* (Ruiz & Pav.) DC.		X	
	*Palicourea angustifolia* Kunth	X	X	
Sapotaceae	*Pouteria caimito* (Ruiz & Pav.) Radlk.	X		
Siparunaceae	*Siparuna echinata* (Kunth) A.DC	X		
Solanaceae	*Solanum* cf. *venosum* Humb. & Bonpl. ex Dunal			X
	*Solanum* sp.			X
Urticaceae	*Cecropia peltata* L.	X		
Viburnaceae	*Viburnum pichinchense* Benth.	X		
	*Viburnum triphylum* Benth	X		
Winteraceae	*Drimys granadensis* L.f.	X		

The letter X indicates the presence in land use.

**Table 2:** Forestal structure and to the plant covers.

	Plant Cover		
Variables	RF	ER	NR
Basal area/ha (m^2^)	15.75 ± 0.03	16.33 ± 0.02	6.08 ± 0.04
No. trees/ha (DAP > 0.1 m)	1138 ± 0.04	1085 ± 0.05	873 ± 0.08

Values are means ± standard deviation calculated across six transects per land-cover type (50 × 4 m; 200 m^2^ each; total sampled area = 0.12 ha per cover). RF: riparian forest; ER: ecological restoration; NR: natural regeneration.

**Table 3:** Diameter distributions of vegetation cover in the studied plots. RF, ER, NR.

Plant Cover	Diameter Classes	DBH Range (m)	Individuals Proportion (%)
RF	I	0.1–0.199	42
	II	0.2–0.299	33
	III	0.3–0.399	15
	IV	0.4–0.499	10
ER	I	0.1–0.199	48
	II	0.2–0.299	30
	III	0.3–0.399	15
	IV	0.4–0.499	7
NR	I	0.1–0.199	65
	II	0.2–0.299	25
	III	0.3–0.399	7
	IV	0.4–0.499	3

**Table 4:** *Altimetric* distributions of vegetation cover in the studied plots. RF, ER, NR.

Plant Cover	Altimetric Classes	Height Range (m)	Individuals Proportion (%)
RF	I	1–4.99	34
	II	5–9.99	40
	III	10–14.99	18
	IV	15–19.99	8
ER	I	1–4.99	42
	II	5–9.99	37
	III	10–14.99	15
	IV	15–19.99	6
NR	I	1–4.99	55
	II	5–9.99	33
	III	10–14.99	9
	IV	15–19.99	3

**Table 5:** Ecological indices by plant cover.

Plant Cover	Shannon	Simpson
RF	2.448895	0.8625982
ER	2.033108	0.7933454
NR	2.890306	0.9176203

## Data Availability

The data that support the findings of this study are available from the corresponding author upon reasonable request. No publicly archived datasets were generated or analyzed during this study.

## Abbreviations

The following abbreviations are used in this manuscript: AFP (Álvaro Fernández Pérez Herbarium), CBH (Circumference at Breast Height), DBH (Diameter at Breast Height), ER (Ecological Restoration), H′ (Shannon–Wiener Diversity Index), NR (Natural Regeneration), PAST (Paleontological Statistics Software), POWO (Plants of the World Online), RF (Riparian Forest), SD (Standard Deviation), UTM (Universal Transverse Mercator), WFO (World Flora Online).
